# Vitamin D and Sarcopenia: Implications for Muscle Health

**DOI:** 10.3390/biomedicines13081863

**Published:** 2025-07-31

**Authors:** Héctor Fuentes-Barría, Raúl Aguilera-Eguía, Lissé Angarita-Davila, Diana Rojas-Gómez, Miguel Alarcón-Rivera, Olga López-Soto, Juan Maureira-Sánchez, Valmore Bermúdez, Diego Rivera-Porras, Julio Cesar Contreras-Velázquez

**Affiliations:** 1 Vicerrectoría de Investigación e Innovación, Universidad Arturo Prat, Iquique 1100000, Chile; hefuentes_@unap.cl; 2 Escuela de Odontología, Facultad de Odontología, Universidad Andres Bello, Concepción 3349001, Chile; 3 Departamento de Salud Pública, Facultad de Medicina, Universidad Católica de la Santísima Concepción, Concepción 3349001, Chile; raguilerae@ucsc.cl; 4 Escuela de Nutrición y Dietética, Facultad de Medicina, Universidad Andres Bello, Concepción 3349001, Chile; lisse.angarita@unab.cl; 5 Escuela de Nutrición y Dietética, Facultad de Medicina, Universidad Andres Bello, Santiago 7550000, Chile; diana.rojas@unab.cl; 6 Escuela de Ciencias del Deporte y Actividad Física, Facultad de Salud, Universidad Santo Tomás, Talca 3460000, Chile; mrivera3@santotomas.cl; 7 Facultad de Medicina, Universidad Católica del Maule, Talca 3460000, Chile; 8 Facultad de Salud, Universidad Autónoma de Manizales, Manizales 170017, Colombia; sonrie@autonoma.edu.co; 9 Programa de Doctorado en Educación, Facultad de Educación, Universidad Bernardo O’Higgins, Santiago 7550000, Chile; juan.maureira74@gmail.com; 10 Centro de Investigaciones en Ciencias de la Vida, Facultad de Ciencias de la Salud, Universidad Simón Bolívar, Barranquilla 080022, Colombia; 11 Departamento de Productividad e Innovación, Universidad de la Costa, Barranquilla 080002, Colombia; drivera23@cuc.edu.co (D.R.-P.); jcontrer30@cuc.edu.co (J.C.C.-V.)

**Keywords:** Vitamin D, Vitamin D deficiency, sarcopenia, muscle strength, nutritional physiological phenomena

## Abstract

Sarcopenia is a progressive age-related musculoskeletal disorder characterized by loss of muscle mass, strength, and physical performance, contributing to functional decline and increased risk of disability. Emerging evidence suggests that vitamin D (Vit D) plays a pivotal role in skeletal muscle physiology beyond its classical functions in bone metabolism. This review aims to critically analyze the relationship between serum Vit D levels and sarcopenia in older adults, focusing on pathophysiological mechanisms, diagnostic criteria, clinical evidence, and preventive strategies. An integrative narrative review of observational studies, randomized controlled trials, and meta-analyses published in the last decade was conducted. The analysis incorporated international diagnostic criteria for sarcopenia (EWGSOP2, AWGS, FNIH, IWGS), current guidelines for Vit D sufficiency, and molecular mechanisms related to Vit D receptor (VDR) signaling in muscle tissue. Low serum 25-hydroxyvitamin D levels are consistently associated with decreased muscle strength, reduced physical performance, and increased prevalence of sarcopenia. Although interventional trials using Vit D supplementation report variable results, benefits are more evident in individuals with baseline deficiency and when combined with protein intake and resistance training. Mechanistically, Vit D influences muscle health via genomic and non-genomic pathways, regulating calcium homeostasis, mitochondrial function, oxidative stress, and inflammatory signaling. Vit D deficiency represents a modifiable risk factor for sarcopenia and functional impairment in older adults. While current evidence supports its role in muscular health, future high-quality trials are needed to establish optimal serum thresholds and dosing strategies for prevention and treatment. An individualized, multimodal approach involving supplementation, exercise, and nutritional optimization appears most promising.

## 1. Introduction

Vitamin D (Vit D) has traditionally been recognized for its essential role in the intestinal absorption of calcium and phosphorus, as well as in maintaining bone health [1]. However, recent research has demonstrated that its functions extend beyond mineral metabolism, with relevant pleiotropic effects in the regulation of immunological, endocrine, and metabolic processes [2,3,4]. From an endocrinological perspective, Vit D is considered a prohormone rather than a classical vitamin, which explains its systemic actions across multiple tissues, including skeletal muscle.

In this context, population aging represents one of the major public health challenges of the 21st century, due to the sustained increase in the prevalence of chronic diseases and age-related functional impairments, which significantly impact the quality of life in older adults [5]. Among these conditions, sarcopenia has gained relevance due to its high prevalence and substantial clinical consequences. This progressive and generalized musculoskeletal disorder is characterized by significant loss of muscle mass, strength, and function [6,7,8,9].

Clinically, sarcopenia is associated with fatigue, weakness, impaired balance, and difficulty performing activities of daily living, all of which increase the risk of falls, fractures, and loss of functional independence [10,11,12]. Emerging scientific evidence suggests that insufficient serum Vit D levels may play a key role in its pathophysiology, given Vit D’s involvement in muscle physiology and its potential impact on strength and physical performance [13,14,15,16].

Several studies have reported that Vit D supplementation may be associated with improvements in muscle function and a lower incidence of frailty in older adults. It has even been suggested that doses exceeding those recommended for bone health may contribute to the prevention or attenuation of sarcopenia [13,17,18]. However, findings have been inconsistent, partly due to the lack of consensus regarding optimal serum Vit D levels, as well as interindividual variability in response to supplementation [13,19].

This heterogeneity may stem from differences in study designs, variability in the diagnostic criteria used to define sarcopenia, and the absence of agreement on the optimal cut-off points for Vit D sufficiency [9,10,13,20,21]. Considering this scenario, the present review aims to critically analyze the available scientific evidence on the relationship between serum Vit D levels and sarcopenia in older adults, to identify potential clinical implications and provide a rationale for the development of effective preventive and therapeutic strategies in the context of healthy aging.

## 2. Vit D: Physiology and Metabolism

Vit D is a liposoluble prohormone essential for multiple physiological processes, beyond its classical role in calcium metabolism and bone health. Its relevance to overall homeostasis is linked to its ability to modulate gene expression in various tissues, including skeletal muscle, making it a key factor in the prevention of chronic diseases and functional decline associated with aging [8,22,23].

### 2.1. Cutaneous Synthesis and Dietary Sources

The primary source of Vit D in humans is endogenous cutaneous synthesis induced by ultraviolet B (UVB) radiation from sunlight, which converts 7-dehydrocholesterol into pre-Vit D_3_, subsequently isomerized to Vit D_3_ (cholecalciferol) [1]. This pathway accounts for approximately 80–90% of the total Vit D input in individuals with adequate sun exposure [24]. Additionally, Vit D can be obtained from dietary sources, mainly animal-based foods such as fatty fish, liver, and egg yolks, and to a lesser extent through fortified foods and supplements, in the forms of Vit D_2_ (ergocalciferol) and Vit D_3_ [25,26].

Once absorbed or synthesized, Vit D is transported to the liver, where it undergoes hydroxylation by the enzyme 25-hydroxylase (CYP2R1), forming 25-hydroxyvitamin D [25(OH)D], considered the major circulating form and the most reliable biomarker of Vit D status [19,27,28]. Subsequently, in the kidneys and other extra-renal tissues such as skeletal muscle and the immune system, a second hydroxylation is carried out by the enzyme 1α-hydroxylase (CYP27B1), producing the biologically active form 1,25-dihydroxyvitamin D [1,25(OH)_2_D], also known as calcitriol [27,28].

### 2.2. Vitamin D Receptors (VDRs) in Skeletal Muscle

The discovery of VDRs in muscle cells has highlighted the direct role of this hormone in skeletal muscle physiology [29]. VDRs are nuclear transcription factors that, upon binding with calcitriol, modulate the expression of genes involved in cell proliferation, differentiation, and function. The presence of these receptors has been confirmed in both myoblasts and mature muscle fibers, suggesting that Vit D may exert autocrine/paracrine actions within muscle tissue [29,30].

From a molecular perspective, the Vitamin D receptor (VDR) is part of the nuclear receptor superfamily. It has a conserved structure with a DNA-binding domain that recognizes VDREs, and a ligand-binding domain that binds calcitriol (1,25(OH)_2_D_3_) [1]. Upon binding, VDR changes conformation and forms a heterodimer with the 9-cis-retinoic acid receptor (RXR). This complex moves to the nucleus and regulates genes related to cell cycle, metabolism, calcium signaling, and muscle contraction [31].

In addition to its genomic effects, VDR can also activate non-genomic signaling pathways by modulating intracellular cascades such as mitogen-activated protein kinase (MAPK), phosphoinositide 3-kinase/protein kinase B pathway (PI3K/Akt), and protein kinase C (PKC), suggesting a rapid and multifaceted influence of Vit D on skeletal muscle homeostasis [32,33,34]. These actions may play a key role in the acute regulation of muscle excitability, insulin sensitivity, and protection against oxidative damage [32,33,34].

**Figure 1:**
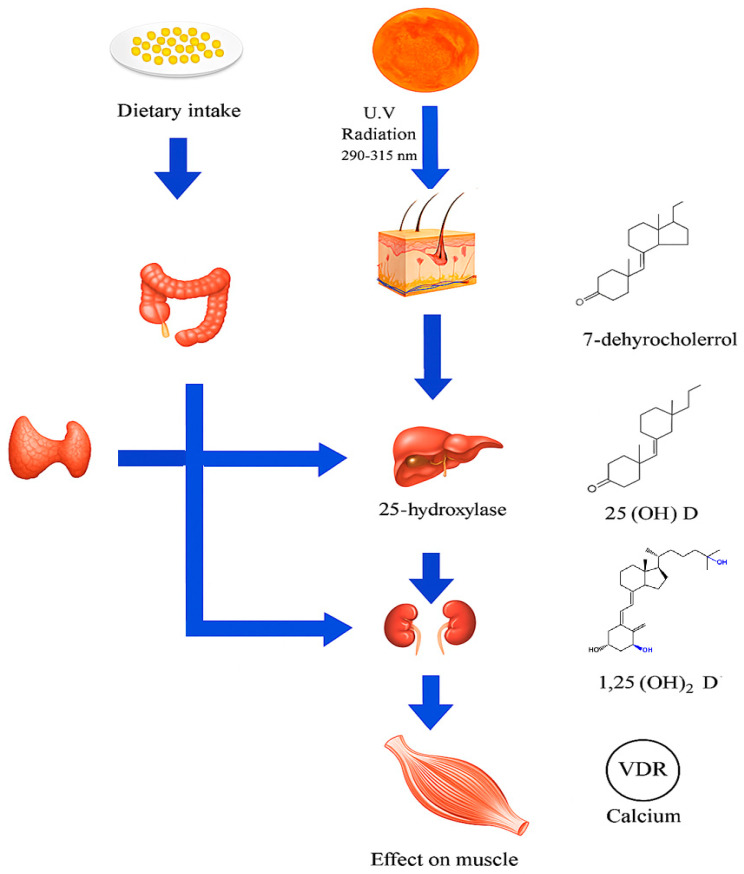
Vit D metabolism in sarcopenia. Source: adaptation of Fuentes-Barría et al. [38].

Furthermore, animal models with muscle-specific VDR gene deletion have shown altered muscle morphology, reduced strength, and changes in the expression of genes related to mitochondrial metabolism and calcium homeostasis. These findings reinforce the notion that VDR-mediated signaling is essential for the structural and functional integrity of skeletal muscle [35,36,37]. Therefore, the study of VDR in muscle tissue not only provides new insights into muscle physiology but also offers important therapeutic implications in the context of muscle disorders, sarcopenia, and physical rehabilitation (Figure 1).

## 3. Sarcopenia

### 3.1. Definition, Diagnostic Criteria, and Pathophysiology

Sarcopenia is a progressive and generalized musculoskeletal syndrome characterized by the loss of muscle mass, strength, and physical performance. It is associated with adverse outcomes such as falls, functional disability, institutionalization, and mortality in older adults. Although it was initially regarded as a physiological consequence of aging, it is now recognized as a complex nosological entity that may occur as a primary condition (age-related) or as secondary to chronic diseases, malnutrition, physical inactivity, or systemic inflammatory processes [10,39].

In response to the need to unify and operationalize its diagnosis, several international working groups have proposed specific criteria. Among them, in 2018, the European Working Group on Sarcopenia in Older People 2 (EWGSOP2) issued a widely adopted redefinition, notable for its functional and stepwise approach. According to this framework, the diagnosis of sarcopenia begins with the detection of reduced muscle strength as the primary criterion; confirmation requires concomitant low muscle mass, and the addition of poor physical performance indicates severe sarcopenia [20]. This hierarchical model facilitates clinical stratification and the design of interventions based on the severity of functional impairment.

The diagnostic methods recommended by EWGSOP2 include handgrip strength testing (cut-offs: <27 kg for men and <16 kg for women), dual-energy X-ray absorptiometry (DXA) or bioelectrical impedance analysis (BIA) to estimate the appendicular skeletal muscle mass index (ASMI), and physical performance tests such as gait speed (<0.8 m/s), the Short Physical Performance Battery (SPPB ≤ 8 points), or the chair stand test (>15 s) [20,40,41,42].

In Asia, the Asian Working Group for Sarcopenia (AWGS) updated its own guidelines in 2019, adapting them to the anthropometric and functional characteristics of the Asian population [21]. While maintaining the same three-dimensional framework (mass, strength, performance), the AWGS established adjusted cut-off points. For instance, handgrip strength thresholds are higher than those used in Europe (<28 kg for men and <18 kg for women), and a gait speed < 1.0 m/s is considered an early marker of functional decline. Moreover, AWGS emphasizes the use of the SPPB (≤9 points) and the Timed Up and Go test (TUG ≥ 12 s) as complementary measures for identifying functional risk.

The Foundation for the National Institutes of Health (FNIH) Sarcopenia Project, based on statistical analyses of aging populations in the United States, proposed a definition focused on low muscle strength combined with muscle mass adjusted for body mass index (BMI). Suggested thresholds include handgrip strength < 26 kg for men and <16 kg for women, and an ASM/BMI ratio < 0.789 for men and <0.512 for women. Although this approach aims to maximize the prediction of disability and functional outcomes, it has been criticized for its complexity and limited applicability across diverse populations [43].

Lastly, the International Working Group on Sarcopenia (IWGS), in its 2011 consensus, established the diagnosis of sarcopenia based on low muscle mass (ASMI < 7.23 kg/m^2^ in men and <5.67 kg/m^2^ in women, measured by DXA) in combination with gait speed less than 1.0 m/s [44]. While muscle strength is not a mandatory component in this model, it is recognized as a valuable functional parameter.

Collectively, these diagnostic frameworks reflect both advancements in the understanding of sarcopenia and the ongoing challenges related to population-specific, methodological, and clinical variability that hinder global standardization. Table 1 summarizes the key similarities and differences among the EWGSOP2, AWGS, FNIH, and IWGS criteria, offering a comparative framework essential for clinical and research practice.

### 3.2. Diagnostic Components: Muscle Mass, Strength, and Physical Performance

Muscle strength has assumed a central role as the primary criterion for the identification of sarcopenia. It reflects the capacity of the neuromuscular system to generate mechanical tension and is influenced by muscle mass as well as neurophysiological, metabolic, and hormonal factors [45,46,47].

Its assessment is primarily conducted through handgrip dynamometry, a simple, reproducible, and highly sensitive test for detecting functional decline. According to EWGSOP2 cut-off values, handgrip strength below 27 kg for men and 16 kg for women is considered indicative of probable sarcopenia [48]. Reduced handgrip strength is progressively associated with aging, with an accelerated decline beginning in midlife and continuing into old age [49]. This decline has been linked to increased risk of falls, loss of independence, institutionalization, recurrent hospitalizations, and all-cause mortality [50]. Therefore, muscle strength is regarded as a key marker of biological aging and functional reserve in both clinical practice and epidemiological research [51].

In parallel, physical performance represents the overall functional expression of the musculoskeletal system during daily activities [52]. Unlike mass and strength, performance integrates dynamic and complex aspects of motor control, including neuromuscular coordination, cardiovascular efficiency, joint function, and central nervous system responsiveness [53,54]. Commonly used tests include gait speed, the Short Physical Performance Battery (SPPB), the Timed Up and Go (TUG) test, and the five-time sit-to-stand test [55,56]. Gait speed, particularly measured over a four-meter distance at usual pace, has emerged as a biomarker of health and longevity [57]. A value below 0.8 m/s is associated with a higher risk of adverse events and is considered a valid threshold for the diagnosis of severe sarcopenia [58].

The SPPB combines three subtests—balance, gait speed, and chair rise—and provides a global score from 0 to 12 points, with values ≤ 8 indicating impaired function. This tool has shown excellent predictive capacity for identifying frailty, disability, and institutionalization [59]. Similarly, the TUG test, which measures the time needed to rise from a chair, walk three meters, turn around, and return to sit down, allows for rapid assessment of fall risk and general functional status [55,60].

It is important to emphasize that these components should not be assessed in isolation. The integrated interpretation of muscle mass, strength, and performance enables accurate classification of sarcopenia stage (probable, confirmed, or severe), which facilitates clinical decision-making [20,21,43,44]. For instance, in the EWGSOP2 framework, reduced muscle strength constitutes the initial diagnostic threshold (“probable sarcopenia”); confirmation requires evidence of low muscle mass. When low physical performance is also present, the diagnosis is classified as “severe sarcopenia,” which implies greater urgency for intervention and follow-up [20,43,44].

Furthermore, the choice of tests and cut-off values must consider factors such as age, sex, ethnicity, and clinical context. In Asian populations, for example, threshold values for muscle mass and strength are lower than those observed in European populations, leading the Asian AWGS to propose adapted criteria [21].

In summary, the three diagnostic components of sarcopenia—muscle mass, strength, and physical performance—offer a multifactorial view of the individual’s musculoskeletal health. Their combined assessment allows for both the detection of sarcopenia and the estimation of its functional impact and prognosis, thereby supporting the implementation of preventive, therapeutic, and rehabilitative strategies tailored to each patient’s specific needs [20,43,44].

### 3.3. Pathophysiology and Etiological Factors: An Integrative Approach

Sarcopenia results from a complex interplay between biological aging and various pathological factors. Among the most relevant pathophysiological mechanisms are chronic low-grade inflammation, anabolic resistance, oxidative stress, mitochondrial dysfunction, and hormonal alterations (e.g., reductions in testosterone, growth hormone, and IGF-1). These changes contribute to decreased protein synthesis and increased muscle catabolism [46,61,62,63].

Physical inactivity is one of the most significant triggers and perpetuating factors, particularly in the context of hospitalization, immobilization, or sedentary lifestyles [64]. In addition, multiple comorbidities such as heart failure, chronic obstructive pulmonary disease, cancer, and type 2 diabetes act as catalysts for the sarcopenic process by promoting pro-inflammatory states or inducing anorexia and cachexia [11,65,66].

Nutritional deficiencies—particularly of protein, Vit D, antioxidants, and omega-3 fatty acids—exacerbate muscle deterioration [67]. Suboptimal protein intake (<1.2 g/kg/day in older adults) limits stimulation of myofibrillar protein synthesis, while Vit D deficiency has been associated with reduced muscle strength and an increased risk of falls. Protein quality, especially the supply of essential amino acids like leucine, is also a crucial factor [68,69].

The progression and clinical expression of sarcopenia vary significantly by sex, age, and ethnicity [70,71]. In general, men tend to have greater absolute muscle mass, but women experience a more rapid functional decline, especially after menopause due to decreased estrogen levels. This hormonal difference may partly explain the higher prevalence of severe sarcopenia in older women, despite lower baseline muscle mass [72,73].

Age is the most strongly associated factor in sarcopenia’s development. From age 50, muscle mass is estimated to decline by 1–2% annually, and this rate may accelerate in the presence of chronic diseases or immobility. Among adults over 80 years of age, prevalence exceeds 40%, emphasizing the need for early interventions [74,75,76]. Ethnic differences are also notable: studies have shown that African populations tend to have greater muscle mass, while Asians present lower values, even at younger ages. These disparities underscore the need for population-specific cut-off values and culturally adapted diagnostic strategies [77,78].

## 4. Relationship Between Vit D and Sarcopenia

Sarcopenia has been associated with several endocrine factors, among which Vit D deficiency stands out [15]. Beyond its classical role in bone metabolism, Vit D has been shown to participate in key muscle processes, including protein synthesis and mitochondrial function [79,80]. Various lines of research have explored this relationship from observational, clinical, and even mechanistic perspectives.

**Figure 2:**
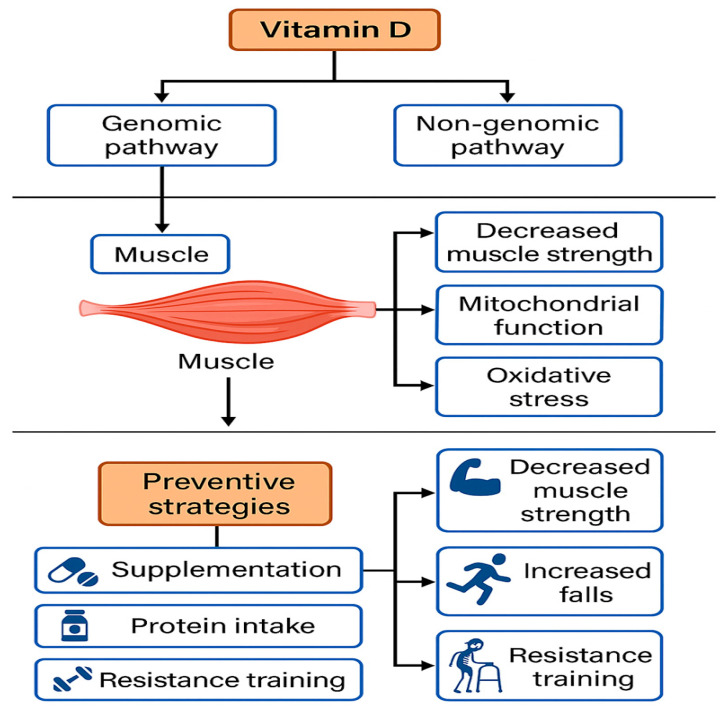
Conceptual model of the Vit D–muscle–sarcopenia axis, illustrating genomic and non-genomic pathways, their effects on muscle function, and preventive interventions.

Recent observational studies have documented a significant association between low serum 25-hydroxyvitamin D (25(OH)D) levels and reduced muscle mass, handgrip strength, and physical performance in older adults. These individuals appear to be at increased risk of developing sarcopenia, even after adjusting for age, physical activity, and comorbidities [81,82]. Such findings have been replicated across diverse populations and have driven the development of preventive strategies in at-risk groups [83]. (Figure 2).

Clinical intervention studies have assessed the impact of Vit D supplementation on muscle-related outcomes. While some randomized controlled trials have reported modest improvements in strength or muscle mass following supplementation with 800 to 2000 IU/day over three months, the results remain inconclusive [84,85]. The response to supplementation seems to depend on multiple factors, including baseline Vit D status, administered dose, concurrent physical activity, and sex [19,38,86]. In general, the effects are more pronounced in individuals with severe deficiency at baseline.

From a pathophysiological standpoint, several plausible mechanisms support the role of Vit D in muscle function [38,86,87,88]. At the molecular level, Vit D modulates the expression of genes involved in muscle protein synthesis and satellite cell proliferation [89]. It also regulates intracellular calcium homeostasis—critical for muscle contraction—and enhances mitochondrial biogenesis, promoting energy efficiency [90,91]. Furthermore, Vit D exerts antioxidant and anti-inflammatory effects that may help mitigate oxidative damage associated with muscle aging [92].

Recent meta-analyses have attempted to clarify these inconsistencies. Some conclude that Vit D supplementation improves muscle strength, but not necessarily muscle mass or physical performance, while others report limited or clinically negligible effects [6,87,88,93,94,95,96]. These discrepancies are partially explained by methodological heterogeneity across studies, including differing diagnostic criteria for sarcopenia, variability in dosage, supplementation duration, and study populations.

Overall, the evidence suggests that Vit D plays an important role in muscle health; however, its therapeutic efficacy in sarcopenia remains to be fully elucidated. Well-designed studies are needed—particularly those targeting specific subgroups and combining supplementation with exercise and nutritional strategies—to better determine its potential benefits [20,21,43,44]. (Table 2).

### 4.1. Vit D Levels

Serum Vit D levels are commonly reported in ng/mL or nmol/L, using 25-hydroxyvitamin D [25(OH)D] measurement as the reference standard. However, there is no universally accepted criterion to clearly define serum sufficiency, insufficiency, or deficiency cut-off points [19]. Commonly accepted thresholds, according to the United States Institute of Medicine (IOM), classify severe deficiency as 10–12 ng/mL (25–30 nmol/L), mild deficiency below 20 ng/mL (<50 nmol/L), and adequate status above 20 ng/mL (>50 nmol/L) [13].

This lack of consensus has generated discrepancies among various clinical guidelines and recommendations, including those issued by the IOM, the International Osteoporosis Foundation, and the American Geriatrics Society. Such divergences reflect not only differences in evidence interpretation but also methodological limitations in measurement protocols, variability in studied populations, and a historically bone-centric focus. Nevertheless, recent studies suggest that optimal levels for muscle function may lie at higher thresholds, especially in older adults, institutionalized individuals, or those at risk of sarcopenia [13,19,87,88,97].

Evidence indicates that levels above 30 ng/mL (75 nmol/L) could be more effective in maintaining muscle mass and strength, as well as preventing progression toward sarcopenia and frailty. This hypothesis is supported by observational data and clinical trials showing higher sarcopenia prevalence in individuals with Vit D insufficiency and modest supplementation benefits in those with severe deficiency [98,99].

Vit D deficiency represents a widespread public health concern, particularly in populations with limited sun exposure (high latitudes) or genetic characteristics reducing cutaneous synthesis, as observed in individuals with darker skin pigmentation. These conditions may compromise endogenous Vit D production and contribute to sustained suboptimal levels, which have significant repercussions on muscle health, functional balance, and fall risk—factors central to sarcopenia pathogenesis [100,101,102].

Table 3 presents a summary of commonly used ranges to categorize Vit D status based on serum 25(OH)D levels. It also highlights the utility of these measures as markers to identify individuals at risk of functional impairment related to sarcopenia and to guide preventive strategies, including supplementation, dietary modification, and promotion of appropriate physical activity.

### 4.2. Recommendations for Vit D Intake

Vit D plays essential roles not only in bone metabolism but also in regulating muscle contraction, cell proliferation, and immunometabolic balance. Adequate intake is particularly important for individuals at higher risk of muscle deterioration, such as older adults, patients with chronic diseases, or those living in regions with limited sun exposure.

At latitudes with low UVB radiation, Vit D deficiency tends to increase during winter months. In these settings, food fortification policies (such as enrichment of dairy products or oils), combined with seasonal preventive supplementation, have demonstrated efficacy in controlling hypovitaminosis D. Ensuring adequate Vit D status in these contexts is critical to prevent functional decline and progression toward frailty and sarcopenia [13,98,103]. Table 4 shows the recommended duration of sun exposure for individuals with light skin on the face and arms without sunscreen, adapted from the Australian Sports Commission. These recommendations vary by region and season, reflecting differences in UVB radiation availability. Notably, individuals with highly pigmented skin require 3 to 6 times longer exposure to achieve comparable Vit D synthesis.

Daily Vit D requirements vary according to life stage and health status. For older adults, especially those over 70 years of age, an intake of at least 20 µg daily (equivalent to 800 IU) is recommended to counteract the age-related decline in cutaneous synthesis and to contribute to the preservation of muscle mass and physical function [13,103]. Multiple international organizations agree that this population group requires higher serum 25(OH)D levels to prevent sarcopenia and its clinical consequences, including falls, disability, and loss of independence [19].

Individuals at increased risk of deficiency, such as those with malabsorption disorders, obesity, chronic kidney disease, or prolonged use of medications like corticosteroids, are advised to receive tailored supplementation ranging from 1000 to 2000 IU daily, depending on baseline plasma levels. In certain clinical scenarios and under medical supervision, higher doses—up to 10,000 IU/day—may be considered to achieve adequate concentrations and sustain beneficial effects on muscle tissue [13].

Conversely, excessive supplement intake poses an increasing risk due to easy access to high-concentration products. Currently, the upper safe daily intake limit for adults is set at 4000 IU, based on available evidence [99]. Chronic use of doses above this threshold can cause adverse effects such as hypercalcemia and hypercalciuria. Furthermore, high single bolus doses (≥300,000 IU) have been associated with increased toxicity risk and are not recommended as routine practice [37,86]. For patients on prolonged supplementation, periodic monitoring of serum Vit D and calcium levels is recommended, with dose adjustments based on individual response and clinical profile.

Table 5 presents an updated summary of daily Vit D intake recommendations according to age and clinical conditions associated with sarcopenia risk. It also includes specific guidance for individuals with metabolic comorbidities, such as diabetes or obesity, in whom Vit D may play a complementary role in regulating insulin sensitivity and preserving lean body mass. The discrepancies among guidelines underscore the importance of considering individual needs, geographic context, and overall health status when defining prevention and supplementation strategies.

## 5. Controversies and Limitations of Current Evidence

Despite the growing body of literature supporting a potential relationship between Vit D and muscle health, significant controversies persist that limit the generalizability of findings and the formulation of consistent clinical recommendations.

One of the main sources of inconsistency among studies is the heterogeneity in supplementation protocols. There are substantial differences in dosages used (ranging from 400 to 4000 IU/day), duration of interventions (from weeks to over a year), and the form of Vit D employed, with cholecalciferol generally being more effective than ergocalciferol in raising serum 25(OH)D levels [105,106]. These variables directly impact studies’ ability to detect significant effects on muscle mass, strength, or functional performance.

Likewise, the diagnostic criteria for sarcopenia used in literature vary widely, complicating comparisons across studies. While some investigations rely exclusively on muscle mass cutoffs (e.g., assessed by DXA or BIA), others incorporate functional components such as handgrip strength or gait speed, in accordance with the EWGSOP or AWGS consensus definitions [20,21,43,44]. This methodological diversity produces divergent results even for similar interventions.

From a biological standpoint, individual differences in response to Vit D represent another critical factor. Elements such as genetic variability, polymorphisms in the vitamin VDR, gastrointestinal conditions affecting intestinal absorption, and baseline nutritional status can significantly influence the bioavailability and efficacy of supplementation. Certain VDR gene variants have been associated with a reduced anabolic response to supplementation, which may explain inconsistent results in apparently homogeneous populations [107,108,109].

Additionally, there is a lack of consensus regarding optimal serum 25(OH)D levels for muscle function. While some authors propose sufficiency thresholds above 30 ng/mL, others consider levels of 20 ng/mL adequate, impacting inclusion criteria and interpretation of results in clinical trials [19].

Finally, important methodological limitations exist in the assessment of muscle parameters. Muscle mass measurement by indirect methods such as bioelectrical impedance analysis (BIA) shows lower precision compared to dual-energy X-ray absorptiometry (DXA), especially in older adults with dehydration or altered body composition. Similarly, although handgrip strength is a simple and validated tool, it does not always reflect total body strength, and results may be influenced by joint conditions or motivational factors [20,21,43,44].

**Figure 3:**
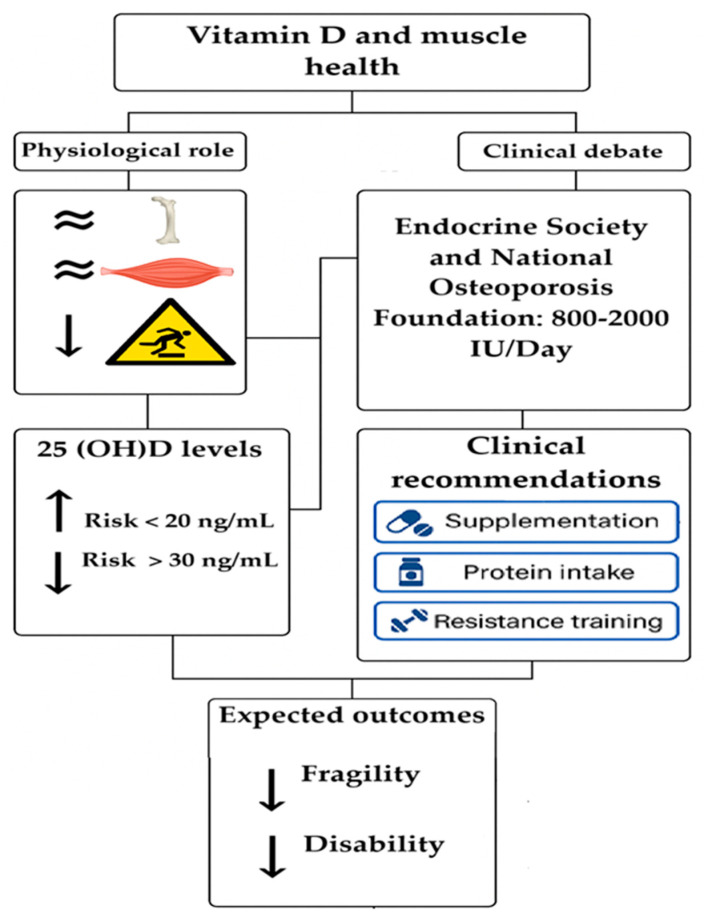
Controversies of current evidence. Source: own elaboration.

Together, these limitations underscore the need for more rigorous, standardized, and long-term clinical trials that consider specific subgroups, genetic biomarkers, and combined strategies involving physical exercise and personalized nutrition to clarify the true potential of Vit D in the prevention and treatment of sarcopenia. Nonetheless, caution should be exercised when interpreting these findings, as several studies included in the present review varied in terms of methodological rigor, population characteristics, and definitions of sarcopenia or functional decline. This heterogeneity may limit the generalizability of the conclusions and underscores the need for more standardized research protocols. Moreover, although positive associations between 25(OH)D levels and physical function are observed, causality cannot be firmly established due to the observational nature of many studies (Figure 3).

Finally, this review itself is subject to certain limitations. The narrative approach, although comprehensive, does not follow a systematic review framework and lacks a formal risk of bias assessment of the included studies. Future research should consider adopting systematic methodologies, including predefined inclusion criteria, structured bias appraisal tools, and meta-analytic synthesis when possible. This would allow for a more robust quantification of the relationship between vitamin D status and sarcopenia and help strengthen evidence-based clinical recommendations.

## 6. Clinical Implications

The potential role of Vit D in muscle health and functional capacity among older adults has sparked considerable debate in geriatric clinical practice [110]. Beyond its classical effects on calcium homeostasis and bone health, numerous studies suggest that maintaining adequate serum levels of 25-hydroxyvitamin D (25(OH)D) may contribute to preserving muscle mass, improving strength, and reducing the risk of falls, frailty, and functional disability [39,42,43].

One of the most pertinent questions is whether systematic Vit D supplementation should be recommended for the elderly population. Current clinical guidelines are not unanimous. While organizations such as the Endocrine Society and the National Osteoporosis Foundation recommend preventive supplementation in older adults at risk of deficiency (doses ranging from 800 to 2000 IU/day), other bodies, like the US Preventive Services Task Force, adopt a more conservative stance due to a lack of conclusive evidence regarding generalized functional benefits. Therefore, decisions should be individualized based on clinical profile, sun exposure, diet, comorbidities, and baseline serum levels [13,111].

Regarding desirable 25(OH)D levels for muscle health, there is partial consensus that concentrations below 20 ng/mL are associated with increased risk of weakness, reduced physical performance, and sarcopenia. However, several studies suggest that optimal levels for muscle function may be above 30 ng/mL, especially in institutionalized older adults or those with chronic illnesses. This threshold remains under debate, and well-controlled trials are needed to specifically define the levels required to optimize muscle function beyond traditional osteometabolic criteria [19].

From a functional perspective, Vit D could play a key preventive role in the frailty cascade by enhancing strength, balance, and neuromuscular responsiveness, which translates into a lower incidence of falls and disability [38,90]. Clinical trials such as PROVIDE have demonstrated that combined supplementation with proteins and leucine can significantly improve appendicular muscle mass and functional performance [112]. Accordingly, combined interventions appear to be the most promising strategy: the synergy between Vit D, high-quality proteins (such as whey), specific amino acids (leucine, HMB), and resistance exercise has been reported to confer superior benefits compared to any isolated intervention [69,112,113].

Consequently, current recommendations should focus on a multifactorial and individualized approach for the prevention of sarcopenia and frailty in older adults. Regular assessment of serum Vit D levels, alongside the promotion of tailored physical exercise (preferably strength training) and a protein-rich diet, represents a low-risk clinical intervention with high potential functional impact. The implementation of comprehensive prevention programs, especially in community or institutional settings, could significantly reduce the burden of disability and improve quality of life in the aging population [20,21,45,46].

## 7. Conclusions

Vit D deficiency constitutes a modifiable risk factor for sarcopenia and functional decline in older adults. This narrative review provides a comprehensive synthesis of the literature linking vitamin D status to muscle health in aging populations. However, it is important to emphasize that much of the available evidence stems from observational studies and interventional trials with significant methodological heterogeneity, which limits the strength and generalizability of the conclusions. This limitation should be explicitly acknowledged to avoid overinterpretation and to guide a more cautious and evidence-informed application in clinical practice. Nonetheless, current data suggest that vitamin D supplementation may be beneficial, particularly in individuals with confirmed deficiency, especially when combined with resistance training and adequate protein intake.

## Figures and Tables

**Table 1:** Comparison of diagnostic criteria for sarcopenia according to EWGSOP2, AWGS, FNIH, and IWGS.

Criteria	EWGSOP2	AWGS	FNIH	IWGS
Operational Definition	I suspected with low muscle strength. Confirmed if low muscle mass is also present. Severe sarcopenia if physical performance is also impaired.	Sarcopenia is diagnosed with low muscle mass plus either low muscle strength or poor physical performance.	Sarcopenia is defined as low muscle strength combined with low muscle mass adjusted for BMI.	Sarcopenia is defined as the presence of low muscle mass and poor physical performance.
Diagnostic Stages	Sarcopenia probably: Low strength. Sarcopenia confirmed: + Low muscle mass. Serve Sarcopenia: + Low physical performance.	Not specified	Not specified	Not specified
Muscle Strength	Handgrip strength: Men: <27 kg Women: <16 kg	Handgrip strength: Men: <28 kg Women: <18 kg	Handgrip strength: Men: <26 kg Women: <16 kg	Not required
Muscle Mass	ASMI (DXA or BIA): Men: <7.0 kg/m^2^ Women: <5.5 kg/m^2^	ASMI (DXA or BIA): Men: <7.0 kg/m^2^ Women: <5.7 kg/m^2^	ASM / BMI: Men: <0.78 kg/m^2^ Women: <0.51 kg/m^2^	ASMI (DXA): Men: <7.23 kg/m^2^ Women: <5.67 kg/m^2^
Physical Performance	Gait speed: <0.8 m/s SPPB ≤8 Chair stand test: >15 s	Gait speed: <1.0 m/s SPPB ≤ 9 TUG ≥ 12 s	Not included	Gait speed: <1.0 m/s
Target Population	European adults ≥65 years	Asian adults ≥60 years	U.S. population ≥65 years	Older adults in general

Source: adaptation of Cruz-Jentoft et al. [20], Chen et al. [21], Cruz-Jentoft et al. [43], and Bianchi et al. [44].

**Table 2:** Summary of meta-analyses on the effects of Vit D, protein supplementation, and/or exercise on sarcopenia.

Author (Ref)	Population and Country	Supplementation	Outcomes SMD (95% CI)	Conclusion
Prokopidis et al. [6].	Adults ≥50 years. Europe, North America (United States), Australia and Brazil.	Vit D.	Short Physical Performance −0.23 (−0.40 to −0.06). Handgrip strength −0.07 (−0.70 to 0.55). Timed Up and Go 0.07 (−0.08 to 0.22). Appendicular lean mass 0.06 kg/m^2^ (−0.32 to 0.44). General muscle strength −0.01 (−0.17 to 0.15). General physical performance −0.02 (−0.23 to 0.18).	Vit D did not improve sarcopenia.
Cheng et al. [87].	Older adults ≥ 60 years. Europe (Germany, Italy and Finland), Asia (Taiwan, Japan and China) and America.	Vit D. Protein. Exercise	Handgrip strength 3.86 (0.52 to 7.21). Chair stand time −1.32 (−1.98 to −0.65).	Vit D, protein, and exercise increase strength.
Barbagallo et al. [88].	Older adults ≥ 60 years. United States, Italy and the Netherlands.	Calcifediol.	Gait speed improved 2.500 (1.768 to 3.223). Handgrip strength 0.532 (0.305 to 0.758). Leg extension 0.641 (0.346 to 0.935).	Calcifediol may improve muscle strength.
Nasimi et al. [93].	Older adults ≥ 60 years. Europe, Brazil, United States, United Kingdom, Canada, Australia, China, Korea, Japan and Taiwan.	Whey protein. Vit D.	Physical function including whey protein 0.561 (0.256 to 0.865). Lean mass including whey protein 0.982 (0.228 to 1.736). Physical function including whey protein 1.211 (0.588 to 1.834). Lean mass including whey protein and Vit D 0.993 (0.112 to 1.874). Muscle Strength including whey protein and Vit D 2.005 (0.975 to 3.035). Physical function including whey protein and Vit D 3.038 (2.196 to 3.879).	Whey protein helps frail adults; Vit D enhances.
Chang et al. [94].	Older adults ≥ 60 years. Europea (Belgium, Germany, Ireland, Italy and Sweden) and United Kingdom.	Whey protein. Leucine. Vit D.	Appendicular muscle mass global 0.27 (0.09 to 0.44). Appendicular muscle mass including exercise 0.45 (0.10 to 0.80). Appendicular muscle mass non-exercise 0.21 (0.01 to 0.41). Handgrip strength global 1.03 (−0.10 to 2.16). Handgrip strength including exercise 1.52 (0.62 to 2.41). Handgrip strength non-exercise 0.07 (−0.13 to 0.27). Short Physical Performance Battery global 1.01 (−0.86 to 2.88). Short Physical Performance Battery including exercise 1.97 (1.54 to 2.40). Short Physical Performance Battery non-exercise 0.06 (−0.14 to 0.26).	Whey protein, leucine, Vit D supplementation, and exercise improve muscle.
Gkekas et al. [95].	Older adults ≥ 60 years. Europe (Italy) and Asia (Japan and China).	Vit D (100–1600 IU/day). Plus protein (10−44 g/day).	Handgrip strength 0.38 (0.01 to 0.75). Sit-to-stand time 0.25 (0.06 to 0.43). Skeletal muscle index 0.25 (−0.006 to 0.51). Appendicular muscle mass 0.25 (−0.406 to 0.91)	Vit D plus protein improves strength only.
Tan et al. [96].	Women aged 40 to 60 at menopausal stage. Europe (Spain, Italy, Denmark and Finland), Asia (Thailand, China, Iran, Korea and Japan), America (Canada, United States and Brazil), Oceania (Australia) and Africa (Egypt).	Vit D. Exercise. ≥12 weeks. 3×/week. 60–90 min.	Body mass including exercise 0.232 (0.097 to 0.366). handgrip strength including exercise 0.901 (0.362 to 1.441). Knee extension strength including exercise 0.698 (0.384 to 1.013). Handgrip strength including Vit D 0.303 (0.130 to 0.476).	Exercise and Vit D improve strength.

**Table 3:** Interpretation of serum Vit D levels.

Levels	Netherlands	Institute of Medicine	International Osteoporosis Foundation and American Geriatrics Society	Expert Opinion
Severe Deficiency	10–12 ng/mL 25–30 nmol/L	10–12 ng/mL 25–30 nmol/L	10–12 ng/mL 25–30 nmol/L	10–12 ng/mL 25–30 nmol/L
Slight Deficiency	N/A	<20 ng/mL <50 nmol/L	<30 ng/mL <75 nmol/L	<40 ng/mL <100 nmol/L
Adequate	>10–12 ng/mL >25–30 nmol/L	>20 ng/mL >50 nmol/L	>30 ng/mL >75 nmol/L	>40 ng/mL >100 nmol/L

N/A: Not applicable. Source: adaptation of Herrera-Molina et al. [19].

**Table 4:** Recommended duration of sun exposure for light skin (face and arms, no sunscreen) in Australia according to approximate latitudes.

City (Approximate Latitude)	Summer 10 a.m. or 2 p.m.	Winter 10 a.m. or 2 p.m.	Winter 12 p.m.
Cairns (~16.9° S).	6 to 7 min.	9 to 12 min.	7 min.
Brisbane (~27.5° S).	6 to 7 min.	15 to 19 min.	11 min.
Sydney (~33.9° S).	6 to 8 min.	26 to 28 min.	16 min.
Melbourne (~37.8° S).	6 to 8 min.	32 to 52 min.	25 min.
Hobart (~42.9° S).	7 to 9 min.	40 to 47 min.	29 min.

Source: Australian Institute of Sport [104].

**Table 5:** Vit D intake is recommended in adults and older adults.

Age (years)	Institute of Medicine				Deficiency Risk for the Endocrine Society	
	AI (µg/UI)	EAR (µg/UI)	RDA (µg/IU)	UL (µg/IU)	IU	UL (IU)
19 to 30	N/A	10/400	15/600	100/4000	1500 to 2000	10,000
31 to 50	N/A	10/400	15/600	100/4000	1500 to 2000	10,000
51 to 70	N/A	10/400	15/600	100/4000	1500 to 2000	10,000
>70	N/A	10/400	20/800	100/4000	1500 to 2000	10,000

AI: Adequate Intake, EAR: Estimated Average Requirement, RDA: Recommended Dietary Allowances, UL: Tolerable upper intake level, IU: international units, µg: microgram, N/A: Not applicable. Source: adaptation of Demay et al. [13].

## Data Availability

The data related to this study are available in this article.
